# 

**Published:** 1894-05

**Authors:** 


### TABLE OF CONTENTS.

ORIGINAL COMMUNICATIONS. PAGE

Some of the Causes of Deterioration of Vital Energies of Dentists. By J. N. FARRAR, M.D., D.D.S., New York City . . . . . . . . . . . . . . 289
Eclecticism, the Best Conservative Practice. By DR. CHARLES F. ALLAN, Newburgh, N. Y. . . . . . . . . . . . . . . . . . . . . . . . . 294
Crown- and Bridge-Work. By CECIL P. WILSON, D.M.D. . . . . . . . 303
Porcelain Contours. By GEORGE F. GRANT, D.M.D., Boston . . . . . . 309
American Dentistry in London. By FRANK M. WILKINSON, D.M.D., Boston . . . . . . . . . . . . . . . . . . . . . . . . . . . 313
Amalgam *versus* Gold. By DR. GEORGE A. MILLS, New York . . . . . . 315
A Substitute for Copper Amalgam. By WILLIAM H. TRUEMAN, D.D.S., Philadelphia . . . . . . . . . . . . . . . . . . . . . . . . 319

REPORTS OF SOCIETY MEETINGS.

New York Odontological Society . . . . . . . . . . . . . . . . . . 320
American Academy of Dental Science . . . . . . . . . . . . . . . . 331

EDITORIAL.

A Half-Century of Anæsthesia . . . . . . . . . . . . . . . . . . . 341
Professor W. H. Eames . . . . . . . . . . . . . . . . . . . . . . . 343

OBITUARY.

William Henry Eames, D.D.S . . . . . . . . . . . . . . . . . . . . 344
Resolutions of Respect to Dr. Eames . . . . . . . . . . . . . . . . 345

DOMESTIC CORRESPONDENCE.

Antidote for Arsenic. Correction of Formula . . . . . . . . . . . 346
Case in Practice . . . . . . . . . . . . . . . . . . . . . . . . . 347

NOTES AND COMMENTS . . . . . . . . . . . . . . . . . . . . . . . . 347

CURRENT NEWS.

Midwinter Fair Dental Congress . . . . . . . . . . . . . . . . . . 348
Joint Meeting of the Iowa and Nebraska State Dental Societies . . . . . 349
Change in the Date of the New Jersey July Examinations . . . . . . . . 350
Recent Patents . . . . . . . . . . . . . . . . . . . . . . . . . . 350
Annual Meeting of the Woman's Dental Association . . . . . . . . . 352

